# Towards Structure-Guided Development of Pain Therapeutics Targeting Voltage-Gated Sodium Channels

**DOI:** 10.3389/fphar.2022.842032

**Published:** 2022-01-27

**Authors:** Phuong T. Nguyen, Vladimir Yarov-Yarovoy

**Affiliations:** ^1^ Department of Physiology and Membrane Biology, University of California, Davis, Davis, CA, United States; ^2^ Department of Anesthesiology and Pain Medicine, University of California, Davis, Davis, CA, United States

**Keywords:** sodium channel, peptide toxin, local anesthetic, voltage sensor, pain therapeutic

## Abstract

Voltage-gated sodium (Na_V_) channels are critical molecular determinants of action potential generation and propagation in excitable cells. Normal Na_V_ channel function disruption can affect physiological neuronal signaling and lead to increased sensitivity to pain, congenital indifference to pain, uncoordinated movement, seizures, or paralysis. Human genetic studies have identified human Na_V_1.7 (hNa_V_1.7), hNa_V_1.8, and hNa_V_1.9 channel subtypes as crucial players in pain signaling. The premise that subtype selective Na_V_ inhibitors can reduce pain has been reinforced through intensive target validation and therapeutic development efforts. However, an ideal therapeutic has yet to emerge. This review is focused on recent progress, current challenges, and future opportunities to develop Na_V_ channel targeting small molecules and peptides as non-addictive therapeutics to treat pain.

## Introduction

Voltage-gated sodium (Na_V_) channels play vital roles in initiating and propagating action potentials in excitable cells, conduct pain signals in primary afferents, and emerged as attractive targets for developing non-addictive pain therapeutics (Mulcahy et al., 2019; Alsaloum et al., 2020). The human genome contains nine Na_V_ channel subtypes expressed in various cell types and tissues (Na_V_1.1 to Na_V_1.9) (Catterall et al., 2005). Na_V_1.7, Na_V_1.8, and Na_V_1.9 channels are predominantly expressed in the peripheral nervous system and linked to various pain disorders in humans (Cheng et al., 2021). Genetic and functional studies have identified and validated Na_V_1.7, Na_V_1.8, and Na_V_1.9 channels as targets for pain treatment. This review briefly summarizes the recent progress, current challenges, and discusses the potential use of advanced computational methods for structure-guided development of selective Na_V_ channel inhibitors as therapeutics to treat pain.

## Current Efforts in the Development of Pain Therapeutics Targeting Specific Binding Sites on Na_V_ Channels

**FIGURE 1:**
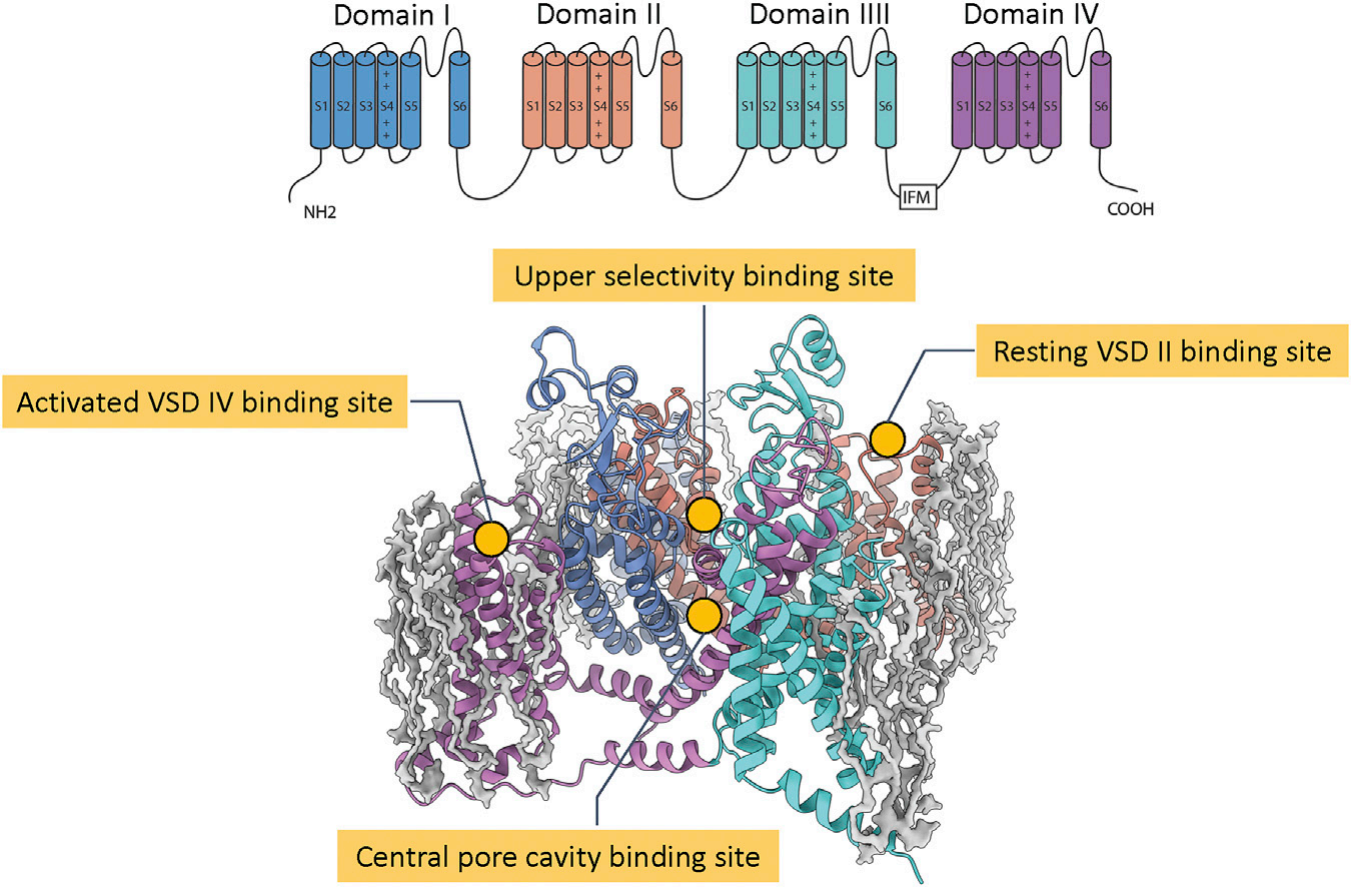
Notable pharmacological binding sites on Na_V_ channels. The top panel shows the transmembrane topology of a eukaryotic Na_V_ channel with four homologous domains and six transmembrane segments S1 to S6. The bottom panel shows a structure of a Na_V_ channel (colored ribbons) with four common pharmacological binding sites (labeled) embedded in a lipid patch (gray blobs).

The structure of eukaryotic Na_V_ channel α subunit comprises four homologous domains I, II, III, IV, each containing six transmembrane segments S1 to S6. The segments S1 to S4 form four distinct voltage sensor domains (VSD I, VSD II, VSD III, and VSD IV) that sense membrane voltage and trigger conformational changes (Ahern et al., 2016) that couple to the pore, which is formed by the segments S5 and S6 from each domain. The membrane depolarization activates VSD I, VSD II, and VSD III to open the channel pore and the subsequent activation of VSD IV leads to the fast inactivation of the channel (Ahern et al., 2016). Biophysical and pharmacological characterization identified multiple binding sites for neurotoxins and drugs on Na_V_ channels (Stevens et al., 2011). Grouping them by location and conformational state, there are four binding sites that have attracted significant interests in the development of pain therapeutics: the central pore cavity, the upper selectivity filter, the resting VSD II, and the activated VSD IV (Figure 1).

### The Central Pore Cavity

**FIGURE 2:**
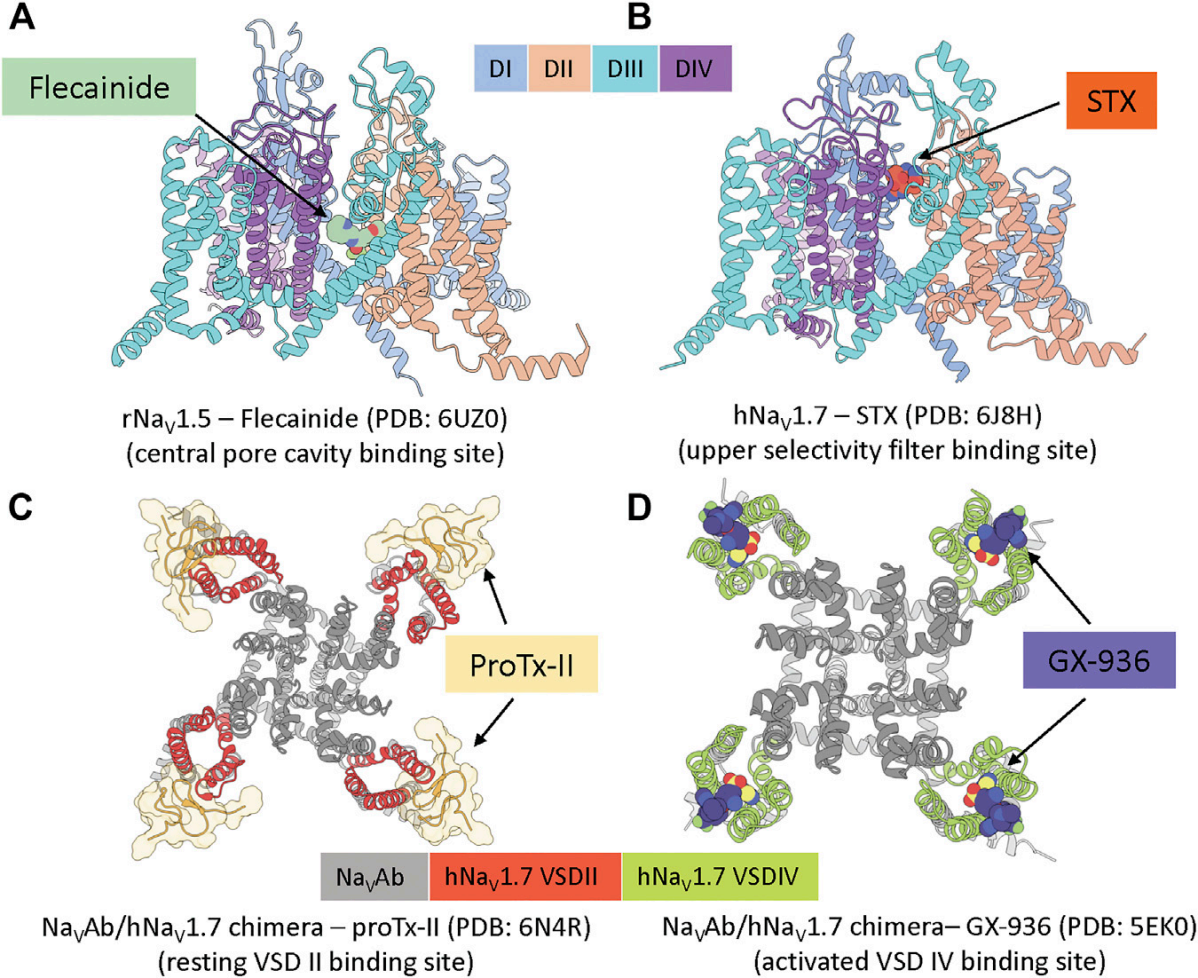
Example structures of small molecules and peptide toxins binding to the four common pharmacological binding sites. **(A)**, rat Na_V_1.5 channel in complex with antiarrhythmic drug flecainide (pdb: 6uz0) (Jiang et al., 2020). **(B)**, human Na_V_1.7 channel in complex with saxitoxin STX (pdb: 6j8h) (Shen et al., 2019). **(C)**, bacterial Na_V_Ab/human Na_V_1.7(VSDII) chimeric channel in complex with protoxin II (pdb: 6n4r) (Xu et al., 2019). **(D)**, bacterial Na_V_Ab/human Na_V_1.7(VSDIV) chimeric channel in complex with investigational compound GX-936 (pdb: 5ek0) (Ahuja et al., 2015).

The central pore cavity binding site is formed by transmembrane segments S5 and S6 (Figure 1). Drugs are thought to access this binding site either from the intracellular side *via* the S6 gate or from the lipid membrane through inter-domain fenestrations (Hille, 1977), although recent evidence has suggested direct access from the extracellular side is possible (Lee et al., 2019; Nguyen et al., 2019). This location is a hub of channel interactions with drugs and neurotoxins. Notably, local anesthetics, such as flecainide (see Figure 2A) and lidocaine, bind to this region and directly block ion conduction or trap the channel in non-conducting states (Hille, 1977). Neurotoxins such as batrachotoxin and veratridine could engage in an interesting dual-action mechanism that can stabilize the open state while partially blocking ion conduction (Wang et al., 2007)^,^ (Craig et al., 2020). Binding at this location often exhibits state-dependent and use-dependent effects in which binding affinity is highly dependent on the membrane potentials and frequencies of stimulation protocols.

This binding site has drawn significant interest due to the clinical use of broad-spectrum small molecule sodium channel inhibitors like local anesthetics, antiarrhythmics, and antiepileptics without adverse effects. For example, FDA has approved lacosamide for partial-onset seizures (Lattanzi et al., 2015), lamotrigine for epilepsy (Nolan et al., 2016), and carbamazepine for the treatment of trigeminal neuralgia (Cruccu et al., 2008). Because these compounds are non-selective, the use cases are limited, highlighting the need for improved therapeutics. However, the high sequence homology among Na_V_ isoforms in the central pore cavity has created challenges for developing selective compounds targeting this site. Investigational compounds such as Vixotrigine (Convergence/Biogen) targeting Na_V_1.7 (Hinckley et al., 2021), currently in phase II clinical trials for trigeminal neuralgia and small fiber neuropathy or PF-01247324 (Pfizer) (Payne et al., 2015), PF-04531083 (Pfizer) (Bagal et al., 2014) targeting Na_V_1.8 are non-selective or have modest selectivity against the on-target channel. Another Na_V_1.8 targeting compound, A-803467 (Abbott/Icagen) also binds to the T-type calcium channel in low micromolar affinity, within the range of therapeutic concentration (Bladen and Zamponi, 2012). Nevertheless, the central pore cavity binding site is still a tractable target even though achieving subtype selectivity could be challenging. Perhaps, this is because broad-spectrum and state-dependent compounds could still be effective and safe for local administrations or conditions with elevated neural activities such as neuropathic pain, as demonstrated by previously approved Na_V_ inhibitor drugs.

### The Upper Selectivity Filter Binding Site

This binding site is located at the entrance of the selectivity filter, mainly formed by residues from the P2 helices in each Na_V_ channel domain (Figure 1). The binding site is well-known for different classes of marine toxins such as tetrodotoxin (TTX), saxitoxin (STX) (see Figure 2B), and μ-conotoxin (Stevens et al., 2011) that directly block sodium ions from passing through the channel. TTX has classically been used to classify Na_V_ channels based on their sensitivity to the toxin: Na_V_1.5, Na_V_1.8, Na_V_1.9 are TTX resistant (TTX-R) while the others are TTX sensitive (TTX-S). Because this binding site is broadly accessible at all membrane potentials, toxin binding here exhibits minimal state-dependent effect, distinguishing this site from all other binding sites mentioned. Mutagenesis and structural studies showed TTX and STX are located deeper in the selectivity filter while μ-conotoxins interact more dominantly with residues at the entrance of the selectivity filter and the extracellular loops of the pore (Walker et al., 2012; Pan et al., 2019). Interestingly, two residues near the binding site for these toxins located on the Na_V_1.7 DIII P2 helix, T1398/I1399 (hNa_V_1.7 numbering), are unique among human Na_V_ channels making this site attractive for subtype selectivity optimization targeting Na_V_1.7.

The development of selective inhibitors for this binding site has been pioneered by SiteOne Therapeutics. Using structure–activity relationship (SAR) study interrogating the T1398/I1399 positions, SiteOne Therapeutics first obtained several selective STX analogs that have >1,000-fold selectivity for Na_V_1.7 over Na_V_1.4 and Na_V_1.6 (Mulcahy et al., 2018). Recently, a compound named ST-2262 was reported to be potent (IC_50_ = 72 nM) with >200-fold subtype selectivity over hNa_V_1.6 and >900-fold over all other hNa_V_ isoforms (except for hNa_V_1.9, which was not tested) (Pajouhesh et al., 2020). ST-2262 was tested on non-human primate pain models and exhibited reduced sensitivity to noxious heat. More recently, the company disclosed another saxitoxin analog named ST-2530 that is potent (IC_50_ = 25 nM) and highly selective with >500-fold over hNa_V_1.1—hNa_V_1.6 and hNa_V_1.8 (Beckley et al., 2021). Testing in mice, the ST-2530 was analgesic in acute pain models using noxious thermal, mechanical, and chemical stimuli when administered subcutaneously. The compound also showed reversal of thermal hypersensitivity after a surgical incision on the plantar surface of the hind paw and transiently reversed mechanical allodynia in the spared nerve injury model of neuropathic pain. Another compound from SiteOne Therapeutics, ST-2427 is currently in Phase I clinical trial.

### The Resting VSD II

The resting VSD II binding site is formed by the extracellular S1-S2 and S3-S4 regions on VSD II (Figure 1). Notably, this site is well-known for binding of several cysteine knot spider toxins, including ProTx-II (see Figure 2C), ProTx-III, HwTx-IV, GpTx-1, and JzTx-V (Schmalhofer et al., 2008; Xiao et al., 2008; Cardoso et al., 2015; Murray et al., 2016; Moyer et al., 2018). These peptide toxins bind with a higher affinity to the resting state of VSD II and can bind with a lower affinity to the activated state of VSD II. High-affinity binding to the resting state of VSD II prevents the voltage sensor from activation upon depolarization and traps the channel in a closed state.

Several peptide toxins targeting resting VSD II binding site are at least partially Na_V_1.7 selective, making them attractive for pain therapeutic developments. However, several reports have pointed out that they have limited analgesic efficacy in animal pain models, likely caused by a narrow therapeutic window (Schmalhofer et al., 2008; Liu et al., 2014; Deuis et al., 2017). The development of pain therapeutics for this binding site focuses on engineering peptides to enhance subtype selectivity targeting Na_V_1.7. Using SAR and multiattribute positional scan (MAPS), Amgen used GpTx-1, a 34-residue tarantula peptide toxin that has potent activity on Na_V_1.7 (IC_50_ = 10 nM) and promising selectivity against important off-target Na_V_ subtypes (20-fold over Na_V_1.4 (skeletal muscle) and 1,000-fold over Na_V_1.5 (cardiac)) to engineer a GpTx-1 analog (named compound 71) that is nearly 10-fold more potent than the wild-type GpTx-1 (IC_50_ = 1.6 nM) and has >1,000-fold selectivity over Na_V_1.4 and Na_V_1.5 (Murray et al., 2015). However, Amgen did not advance this compound for further evaluation *in vivo*. Amgen also used another tarantula peptide toxin, 29-residue JzTx-V, to engineer several highly selective analogs (Wu et al., 2018). The final lead peptide (named compound AM-6120) has six modifications from the wild-type toxin, including three non-canonical amino acids. AM-6120 is highly potent (IC_50_ = 0.8 nM), has ∼ 100 folds selectivity over Na_V_1.4 and >750 folds over Na_V_1.5, Na_V_1.6 and Na_V_1.8. Testing in animal pain models showed that the compound robustly blocked histamine-induced scratching in mice after subcutaneous administration. Notably, high plasma drug concentration (100-fold over the *in vitro* IC_50_) was essential to establish the pharmacological effect. Additionally, AM-6120 had no effect in a capsaicin-induced nociception pain model. The developers suggested that AM-6120 may need high exposure in plasma to achieve effective target entanglement, a problem that has been previously characterized for Na_V_1.7 small molecule inhibitors. Amgen has filed related patents for GpTx-1 and JzTx-V analogs, but no further development has been disclosed.

Using a thorough positional scanning approach, Janssen produced a library of 1,500 analogs of ProTx-II, a highly potent tarantula toxin (IC_50_ < 1 nM) and Na_V_1.7 selective ( >30 folds) to identify the lead compound JNJ63955918 (Flinspach et al., 2017). The compound is less potent (IC_50_ ∼ 10 nM) than the wild-type but has a better selectivity profile, with 100- to >1,000-fold selectivity over Na_V_1.1, Na_V_1.2, Na_V_1.4, Na_V_1.5, and Na_V_1.6. In animal models, central and peripheral administration of JNJ63955918 not only cause a pharmacological insensitivity to chemical and thermal pain and induces itch, which resemble behaviors observed in Na_V_1.7 knock-out in adult mice (Gingras et al., 2014). Janssen has filed a patent for ProTx-II analogs but no further development has been disclosed.

The efforts by Amgen and Janssen were mainly performed with limited structural guidance. Recently, Genentech solved the structure of Na_V_1.7/Na_V_Ab chimera in complex with ProTx-II in resting and activated VSD II states, revealing structural determinants of toxin binding at this site (Xu et al., 2019) (Figure 2C). The structure has revealed the channel–toxin interactions in detail and confirmed the role of F813 (hNa_V_1.7) in the S3 segment of VSD II as important determinant of hNa_V_1.7 selectivity (Schmalhofer et al., 2008). It also revealed a minimum contribution of the S1-S2 region to the interaction with ProTx-II, leaving room for future optimization of peptides targeting this region for subtype selectivity.

### The Activated VSD IV

The activated VSD IV binding site is formed by the extracellular S1-S2 and S3-S4 regions on VSD IV (Figure 1). A considerable development in recent years is the discovery of sulfonamide-based small molecule compounds binding to this site that have resulted in multiple selective inhibitors against Na_V_1.7. These compounds bind to activated conformations of VSD IV and trap the channel in an inactivated state. Since the activation of VSD IV is required, this binding site is available at depolarized membrane potentials, thus creating state-dependent effects of binding.

Pfizer and Icagen first discovered the binding site in the effort that led to the development of selective sulfonamide compounds ICA-121431 and PF-04856264 targeting Na_V_1.3 and Na_V_1.7, respectively (McCormack et al., 2013). PF-04856264 is a state-dependent modulator of Na_V_1.7 with a binding preference for an inactivated state. The potency of PF-04856264 measured with protocols favoring inactivated states was 28 nM for hNa_V_1.7 and >10 μM for Na_V_1.3 and Na_V_1.5, showing promise of this binding site for Na_V_ channel subtype selectivity optimization (McCormack et al., 2013). Pfizer later advanced a subsequently optimized version of PF-04856264 named PF-05089771 into clinical trials. The potency of PF-05089771 was 15 nM for hNa_V_1.7 with >600 fold selectivity over Na_V_1.3, Na_V_1.4, Na_V_1.5, Na_V_1.8, and modest selectivity over Na_V_1.2 ( ∼ 8 folds) and Na_V_1.6 ( ∼ 11 folds) (Swain et al., 2017). PF-05089771 was studied most extensively with multiple clinical trials completed (Mulcahy et al., 2019). Notably, in a phase II randomized, double-blind study for diabetic peripheral neuropathy, PF-05089771 was less effective than the control using pregabalin, a common drug used for the disease and did not show a significant difference to the placebo (McDonnell et al., 2018). In another phase II study to assess the efficacy of increasing oral daily doses of PF-05089771 for the treatment of postoperative dental pain, the compound did not show superiority over ibuprofen which used as a control although adverse effects were similar (US National Library of Medicine, 2021). The predefined efficacy criteria were not meet and these studies did not proceed further. Several studies on similar compounds including PF-06456384 (Pfizer) and AMG8379 (Amgen) suggested that the lack of efficacy is possibly from high binding to plasma proteins, thus leading to low concentrations at the target binding site. Specifically, high unbound plasma exposure relative to *in vitro* potency against Na_V_1.7 was necessary to achieve pharmacodynamic effects in these studies (Kornecook et al., 2017; Pero et al., 2017; Storer et al., 2017; Sun et al., 2019).

More recently, Genentech developed and optimized other sulfonamide compounds to target Na_V_1.7. Notably, Genentech captured the X-ray structure of a Na_V_1.7/Na_V_Ab chimera in complex with sulfonamide, GX-936, revealing the structural basis for sulfonamide interaction with the activated VSD IV binding site (Ahuja et al., 2015) (Figure 2D). The Na_V_1.7/Na_V_Ab—GX-936 structure complex, together with physiological characterizations of another sibling compound, GX-674 on Na_V_1.7 VSD IV, revealed critical roles of R4 gating charge on S4 and YWxxV motif on S2 for potency and selectivity, respectively. Genentech in collaboration with Xenon Pharmaceuticals then advanced two highly potent acyl sulfonamide candidates targeting Na_V_1.7, GDC-0276 (IC_50_ = 0.4 nM) and GDC-0310 (IC_50_ = 0.6 nM) for clinical developments (Safina et al., 2021). GDC-0276has > 21 folds selectivity over hNa_V_1.1, hNa_V_1.2, hNa_V_1.4, hNa_V_1.5, hNa_V_1.6 with the highest selectivity against hNa_V_1.6 ( ∼ 1,200 fold) and the lowest selectivity against hNa_V_1.4 ( ∼ 21 fold). GDC-0310has > 63 fold selectivity over hNa_V_1.1, hNa_V_1.2, hNa_V_1.5, ∼ 330 fold over Na_V_1.6 but only a modest ∼ 6 folds selectivity over hNa_V_1.4 (Safina et al., 2021). Xenon Pharmaceuticals and Genentech have stopped the development of GDC-0276 and GDC-0310 after Phase I trials for non-disclosed reasons.

We are not aware of any related compounds currently in clinical trials, although improvements in minimizing plasma binding have been observed in a next-generation sulfonamide compound such as AMG8379 (Kornecook et al., 2017). Whether optimizing sulfonamide compounds is still a good strategy for developing pain therapeutics remains to be seen. The activated VSD IV binding site, however, has demonstrated promise for developing subtype selective Na_V_ channel inhibitors.

### Undisclosed Binding Site

It is worth to mention that Vertex has developed a series of pyridone amide-based compounds that are selective against Na_V_1.8. The compound VX-150 achieved >400-fold selectivity over other Na_V_ channels (Hijma et al., 2021). Another Vertex compound VX-548 is currently in phase II clinical trials for acute pain after a bunionectomy. However, binding sites for these small molecules have not been revealed yet.

## Potential use of Rosetta in Structural Guided Development of Pain Therapeutics

Designing selective Na_V_ inhibitors as therapeutics to treat pain has been extremely challenging. Only several highly selective compounds have been identified or created by the massive efforts from the biopharmaceutical industry. However, it is noted that a majority of the approach utilized extensive SAR studies in combination with positional scanning and high-throughput testing, whereas high-resolution structural information remains largely unexplored. Since then, many of Na_V_ structures have been solved at high resolution, including in complexes with is small molecules and peptide toxins, providing a better understanding, both structurally and mechanistically of Na_V_ inhibitors at their pharmacological binding sites. Advanced computational methods can take advantage of atomic scale structural information for design and optimization of novel compounds. For example, molecular docking with high-resolution structures led to the identifications of highly selective D_4_ dopamine receptor agonists (Wang et al., 2017) and M3R muscarinic acetylcholine receptor antagonists (Liu et al., 2018). Virtual screening of 490 million virtual molecules discovered selective ligands for sigma 2 receptor (σ_2_R) that showed analgesic effects in animal models for neuropathic pain (Alon et al., 2021). *De novo* protein design functionalities in Rosetta allowed the design of highly potent and selective cytokine mimics (Silva et al., 2019) and picomolar affinity SARS-CoV-2 miniprotein inhibitors (Cao et al., 2020). We expect that high-resolution structures and advanced computational methods will further drive the development of therapeutics targeting Na_V_ channels to treat pain.

Several tools in the Rosetta computational modeling suite may offer promising strategies for the structure-guided approach, both for small molecule and peptide design and optimization. For example, RosettaLigand has been reliable in docking small molecule ligands and is highly suitable for SAR study (Davis and Baker, 2009). The recent integration of the BCL Cheminformatics package (Brown et al., 2021) will soon allow virtual screening and fragment-based design of small molecules in RosettaLigand. Alternatively, a newly developed application for small molecule docking named Rosetta GALigandDock utilizing a fast genetic algorithm would be suited for the virtual screening of small molecule libraries (Park et al., 2021). Rosetta has demonstrated excellent potential to design novel proteins. A large population of peptide inhibitors of ion channels is from natural toxins such as cysteine knot peptides which can now be designed computationally using Rosetta to access more diverse shapes and sizes (Bhardwaj et al., 2016). Such a library of *de novo* designed peptides can then be incorporated with a binding motif on a Na_V_ structure—toxin complex using MotifGraft or FunFolDes (Silva et al., 2016) followed by a sequence design step to achieve novel peptide binders. Additionally, Rosetta also allows the design of peptides with cyclic topologies and with non-canonical amino acids such as macrocycles (Hosseinzadeh et al., 2017). Such approaches would significantly expand peptide compounds’ structural and chemical space to increase the chances of achieving potent and selective inhibitors.

## Conclusion

Human Na_V_ channel subtypes Na_V_1.7, Na_V_1.8, and Na_V_1.9 have emerged as viable targets for developing novel therapeutics to treat pain. Previous efforts by academia and industry have identified small molecules and peptides targeting Na_V_1.7 ad Na_V_1.8 as potential preclinical leads, with several compounds entering clinical trials. We have categorically grouped these efforts into four pharmacological binding sites; each has distinct characteristics that have attracted developments in the biopharmaceutical industry. We envision computational structural biology approaches, such as Rosetta (Baek et al., 2021), AlphaFold (Tunyasuvunakool et al., 2021) and others will be guiding the future design of highly potent and selective Na_V_ inhibitors.

Additionally, advanced mathematical modeling and machine learning methods for absorption, distribution, metabolism, and excretion assessment will further guide the development of next-generation investigational compounds for pain therapeutics.
